# Shining a light on cerebral autoregulation: Are we anywhere near the truth?

**DOI:** 10.1177/0271678X241245488

**Published:** 2024-04-11

**Authors:** Jordan D Bird, David B MacLeod, Donald E Griesdale, Mypinder S Sekhon, Ryan L Hoiland

**Affiliations:** 1 Division of Critical Care Medicine, Department of Medicine, 8167 Faculty of Medicine, Vancouver General Hospital, University of British Columbia, Vancouver, BC, Canada; 2 Collaborative Entity for REsearching BRain Ischemia (CEREBRI), University of British Columbia, Vancouver, BC, Canada; 3 Human Pharmacology & Physiology Lab, Department of Anesthesiology, Duke University Medical Center, Durham, NC, USA; 4 Department of Anesthesiology, Pharmacology & Therapeutics, Faculty of Medicine, The University of British Columbia, Vancouver, BC, Canada; 5 Centre for Clinical Epidemiology & Evaluation, Vancouver Coastal Health Research Institute, Vancouver, BC, Canada; 6 International Collaboration on Repair Discoveries, University of British Columbia, Vancouver, BC, Canada; 7 Djavad Mowafaghian Centre for Brain Health, University of British Columbia, Vancouver, BC, Canada; 8 Division of Neurosurgery, Department of Surgery, Faculty of Medicine, University of British Columbia, Vancouver, BC, Canada; 9 Centre for Heart, Lung and Vascular Health, University of British Columbia, Kelowna, BC, Canada

**Keywords:** Near-infrared spectroscopy, cardiac arrest, cerebral autoregulation, hypoxic-ischemic brain injury, return of spontaneous circulation

## Abstract

The near-infrared spectroscopy (NIRS)-derived cerebral oximetry index (COx) has become popularized for non-invasive neuromonitoring of cerebrovascular function in post-cardiac arrest patients with hypoxic-ischemic brain injury (HIBI). We provide commentary on the physiologic underpinnings and assumptions of NIRS and the COx, potential confounds in the context of HIBI, and the implications for the assessment of cerebral autoregulation.

## Commentary

Near-infrared spectroscopy (NIRS) is utilized in the intensive care and intra-operative settings to continuously and non-invasively monitor regional cerebral oxygen saturation (rSO_2_). Recently, NIRS has been applied to the assessment of cerebral autoregulation in neurocritically ill patients, including post-cardiac arrest patients with hypoxic-ischemic brain injury (HIBI).^1^ With continuous measurements of rSO_2_ and mean arterial pressure (MAP), autoregulation is ostensibly estimated from the moving Pearson correlation coefficient between rSO_2_ and MAP – termed the cerebral oximetry index (COx). A COx value <0.3 is considered to reflect intact autoregulation whereas a value ≥0.3 denotes impaired autoregulation.

Recently, Tachino et al. investigated the association between dysfunctional autoregulation (determined by COx) in HIBI patients and all-cause in-hospital mortality.^2^ In agreement with prior work,^3,4^ they concluded that survival was lower in patients with more time spent with impaired autoregulation and monitoring autoregulation may be useful in the early management of HIBI patients to predict outcome. In contrast, prior work has shown that COx does not differ between patients with good and poor outcome^5^ and poorly agrees with other autoregulation indices (*e.g*., pressure reactivity index; PRx).^1,6^ Therefore, our commentary aims to place the results of Tachino and colleagues into the broader context of monitoring autoregulation as it pertains to 1) physiologic principles of NIRS, and 2) NIRS for monitoring cerebral autoregulation.

### Physiologic principles of cerebral oximetry

Several questions regarding the utility of NIRS as a surrogate measure to characterize physiology require consideration. First, what is the variable/function we truly aim to measure? In the study by Tachino et al., and for a broader neuromonitoring context, the true variable of interest is typically cerebral blood flow (CBF). However, NIRS approximates oxygen saturation by estimating mixed arteriovenous hemoglobin saturation within the cerebral vasculature based on the differing absorptions of oxygenated and deoxygenated hemoglobin. Of note, assorted chromophores (*e.g*., melanin) contribute to the optical properties of the tissue, which impact NIRS measurement beyond just hemoglobin. A second question then becomes, what governs cerebrovascular hemoglobin saturation? Per the Fick equation, cerebrovascular oxygen saturation is determined by cerebral metabolism (CMRO_2_), CBF, and arterial oxygen saturation (or content).

(1) $${\mathrm{CMRO}}_{2}=\mathrm{CBF}\times \left({{\text{C}}_{\text{a}}\text{O}}_{2}-{{\text{C}}_{\text{v}}\text{O}}_{2}\right)$$

where C_a_O_2_ and C_v_O_2_ are arterial and venous oxygen content, respectively. Assuming a constant hemoglobin concentration and arterial oxygen saturation, cerebral venous oxygen saturation (S_v_O_2_) would be the primary determinant of cerebrovascular oxygen saturation and determined as follows:

(2) $${S_{v}O}_{2}\propto \frac{\mathrm{CBF}}{{\mathrm{CMRO}}_{2}}$$

From this relationship, S_v_O_2_ and therefore rSO_2_ are proportional to CBF by virtue of a higher CBF reducing the oxygen extraction fraction (O_2_EF). Conversely, S_v_O_2_ is inversely proportional to CMRO_2_, where a greater metabolic demand would increase O_2_EF and reduce S_v_O_2_. Further, rSO_2_ is influenced by the ratio of arteriole-to-venule blood volume, which is assumed as static when using NIRS.^7^ If CMRO_2_ and the arteriole-to-venule blood volume ratio are constant, rSO_2_ would be expected to be proportional to S_v_O_2_. However, CMRO_2_ is reduced following cerebral ischemia^7^ and the arteriole-to-venule blood volume ratio is not static during alterations in CBF.^8^ These physiologic limitations raise the question of, does rSO_2_ truly reflect CBF? Current evidence suggests that rSO_2_ does not accurately reflect changes in CBF in healthy humans.^1^

**Figure 1.**
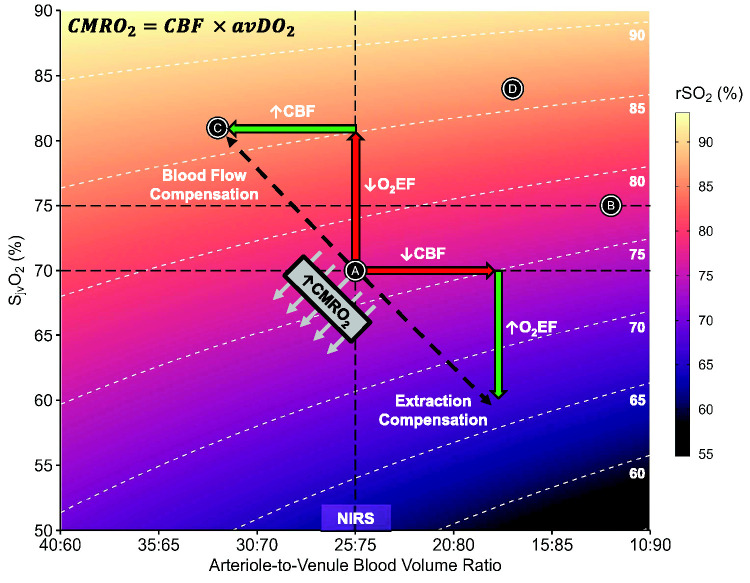
The physiologic underpinnings of cerebral oximetry. This figure depicts the mathematical relationship between S_jv_O_2_ (y-axis), the arteriole-to-venule blood volume ratio (x-axis), and near-infrared spectroscopy derived measures of rSO_2_ (color gradient scale) at a fixed S_a_O_2_ of 98%. rSO_2_ values were computed based on the formula: $rSO_{2}=\left(\left({\%\mathrm{Volume}}_{\mathrm{arterial}}\times S_{a}O_{2}\right)+\left({\%\mathrm{Volume}}_{\mathrm{venous}}\times S_{jv}O_{2}\right)\right)$ . The white dashed lines represent iso lines for values of rSO_2_ between 60 and 90 in 5% increments (labelled in white text). The horizontal dashed black lines denote a normal range for S_jv_O_2_ in healthy humans as reviewed in Hoiland et al., 2023.^7^ The vertical dashed line denotes a typical fixed fraction of arteriole-to-venule blood (25:75) used by NIRS devices. The diagonal black arrows denote equivalent CMRO_2_ that can be achieved by a combination of changes in CBF and O_2_EF – this represents physiologic compensation by CBF or O_2_EF when the other changes. In the case of HIBI, CMRO_2_ is reduced following cerebral ischemia, which shifts the diagonal line up and to the right. Both points A & B as well as C & D, demonstrate how the same rSO_2_ value can originate from a markedly different combination of CBF, O_2_EF, and CMRO_2_ values. This indicates the difficulties of using rSO_2_ as a surrogate for CBF as well as to differentiate between normal physiology and abnormal physiology (i.e., pathophysiology). Note: the range of arterial-to-venous blood ratios presented in this figure fall within the range observed by Ito et al., 2005 during increases and decrease in CBF.^8^ avDO_2_: cerebral arteriovenous oxygen difference; CBF: cerebral blood flow; CMRO_2_: cerebral metabolic rate of oxygen; O_2_EF: cerebral oxygen extraction fraction; COx: cerebral oximetry index; rSO_2_: regional cerebral hemoglobin oxygen saturation; S_a_O_2_: arterial hemoglobin oxygen saturation; S_jv_O_2_: jugular venous hemoglobin oxygen saturation.

Figure 1 highlights the difficulties of interpreting rSO_2_ values. Under normal physiological conditions, points A and C have identical CMRO_2_ despite being achieved by different means. This, however, is not reflected in rSO_2_ where it is lower at point A. Conversely, concurrent reductions in CBF and O_2_EF (point B) have an equivalent rSO_2_ with normal physiology (point A). In HIBI this discrepancy could result from increases in cerebrovascular resistance, impaired diffusion of oxygen from the cerebral vasculature into brain tissue or a pathophysiologic depression in CMRO_2_ following cerebral ischemia,^7^ whereby rSO_2_ would appear normal and be invalid as a CBF surrogate. Therefore, in certain contexts rSO_2_ is unable to differentiate between normal physiology and pathophysiology. This may in part explain the recurrent finding that rSO_2_ is not different between favorable and unfavorable outcomes in HIBI.^2–5^

### NIRS for monitoring cerebral autoregulation

Given the discordance between rSO_2_ and CBF, the next question that arises is, what are the implications for COx as a metric of autoregulation, particularly in HIBI? First, normal jugular S_v_O_2_ is ∼70–75% in healthy subjects^7^ but elevated in a subset of HIBI patients as demonstrated by Sekhon et al.,^9^ and Richter et al.,^10^ suggesting reduced O_2_EF. Under these conditions, the arterial-jugular SO_2_ difference may converge irrespective of CBF (Figure 1), with CBF and oxygen utilization uncoupled.^7^ Indeed, raw rSO_2_ traces show minimal response to changes in MAP in some cases.^1^ As such, COx values approaching zero can suggest either (a) functional autoregulation or (b) impaired cerebral O_2_EF/O_2_ utilization. In this context COx cannot distinguish between functional and impaired autoregulation in HIBI.

Specific to autoregulation metrics, COx lacks agreement and provides conflicting classifications of whether autoregulation is intact compared with other autoregulation indices.^1,6^ Further, readily available moving correlation coefficients have encouraged the simplistic notion of autoregulation being dichotomously present or absent. Such classifications are susceptible to mathematical errors that can arise from impaired O_2_EF^7^ or truncated data. For example, raw MAP signals can have minor fluctuations over time, leading to classification of autoregulation based on minimal change (*e.g*., <2 mmHg). Truncated data confuses steady-state physiology and general signal variability and could explain observations of autoregulation repeatedly appearing then disappearing.^2^

Technological advancements have provided many exciting opportunities to interrogate patient physiology. However, as we strive to shine a light on cerebral autoregulatory function in HIBI patients, indices that are built on variable interactions, physiological assumptions, and indirect measures require scrutiny pertaining to edge cases (pathophysiology), limitations (COx not being comparable to other autoregulation indices), and the complexities (mathematical, physiological) that underpin such metrics.
